# From stiffness to automaticity: visuomotor training alters postural control strategies in older adults

**DOI:** 10.1007/s11357-026-02204-z

**Published:** 2026-03-19

**Authors:** Jakub Malik, Natalia Główka

**Affiliations:** 1 Department of Sports Psychology, Poznan University of Physical Education, Królowej Jadwigi 27/39, 61-871 Poznan, Poland; 2 Department of Sports Dietetics, Poznan University of Physical Education, Królowej Jadwigi 27/39, 61-871 Poznan, Poland

**Keywords:** Juggling, Aging, Dual-task, Bernstein’s theory, Attentional resources, Balance

## Abstract

Aging is often associated with a maladaptive “stiffness” strategy of postural control, which limits adaptability and increases fall risk. Complex visuomotor training (e.g., juggling) may counteract this decline, but the relationship between biomechanical reorganization and cognitive cost reduction remains unclear. We hypothesized that juggling would induce a shift from stiffness to “active monitoring” and reduce the dual-task cost. This exploratory secondary analysis of a randomized crossover trial consists of twenty-five older adults (70 ± 4 years). Participants underwent 4 weeks of juggling training versus a passive control period. Postural sway was assessed using a force platform to analyze kinematic metrics reflecting sway activity (path density) and spatial precision (radial standard deviation). Cognitive cost was assessed via dual-task cost during a counting task. Linear mixed models were used for analysis. Juggling training significantly increased path density (*p* = 0.014) and improved central precision (reduced radial standard deviation, *p* = 0.040). This pattern indicates a transition to an “active monitoring” strategy, effectively “unfreezing” rigid postural control. However, this biomechanical reorganization was not accompanied by a statistically significant reduction in dual-task cost (*p* = 0.08). Visuomotor training effectively “unfreezes” rigid postural strategies in older adults, promoting active, exploratory control. However, these findings should be interpreted as hypothesis-generating. The lack of significant cognitive cost reduction suggests a temporal dissociation: biomechanical flexibility is restored before full automatization occurs, warranting verification in larger, longitudinal studies. The study was retrospectively registered (30.10.2023) at ClinicalTrials.gov (NCT06108713).

## Introduction

Falls represent the leading cause of injury-related morbidity and loss of independence in older adults [1, 2], with age-related declines in postural control serving as a primary contributing factor [3, 4]. A growing body of evidence demonstrates that aging fundamentally alters the neuromuscular strategies employed for balance maintenance [5], with older adults characteristically adopting a “stiffness” strategy marked by heightened agonist-antagonist muscle co-contraction [6, 7]. While this compensatory mechanism confers immediate stability by increasing joint stiffness and limiting excessive excursions during postural perturbations [8, 9], it exacts a substantial cost: reduced adaptability and diminished capacity for dynamic balance adjustments [7, 10]. Falk and colleagues [7] demonstrated that older adults classified as “fallers” exhibited greater reliance on “stiffening” strategies and showed impaired feedforward adaptation across repeated perturbations compared to their non-falling counterparts. Control-theory models corroborate these empirical findings, predicting that age-related increases in sensorimotor delays necessitate compensatory elevations in ankle stiffness via co-contraction, accompanied by coordinated reductions in spinal feedback gain [8]. This represents a fundamental paradox of aging postural control: the very mechanism that prevents acute instability—muscular co-contraction—simultaneously constrains the flexible, context-sensitive adjustments required for effective responses to unexpected environmental challenges [7, 11]. Biomechanical investigations further reveal that older adults exhibit reduced Achilles tendon stiffness coupled with larger muscle fascicle shortening during standing, parameters that correlate with increased center of pressure (CoP) velocity and trembling [12]. These findings suggest that peripheral musculotendinous alterations compound the central nervous system’s reliance on co-contraction strategies [12], creating a multi-level constraint on postural adaptability [10].

Given the adaptability deficits imposed by stiffness-dominant control strategies [7], interventions targeting neuroplasticity and motor relearning have emerged as promising countermeasures to age-related balance decline [13, 14]. Complex visuomotor training paradigms—exemplified by juggling—represent particularly compelling approaches due to their intensive demands on sensorimotor integration, spatial-temporal coordination, and adaptive motor planning [15, 16]. Juggling requires continuous visual tracking, predictive control, bilateral coordination, and real-time error correction [17], thereby engaging distributed neural networks implicated in both motor execution and cognitive-motor integration [18]. The theoretical rationale for such interventions rests on established principles of experience-dependent neuroplasticity, whereby repeated exposure to challenging sensorimotor tasks can induce structural and functional reorganization in aging brains [15, 19].

However, interpreting post-intervention improvements requires careful consideration of motor learning theory, which distinguishes between transient “arousal” effects and enduring “motor learning” effects [20]. Arousal effects—immediate, short-lived performance enhancements driven by heightened attention, motivation, or physiological activation—may manifest as acute improvements in balance metrics during or immediately following training sessions, yet dissipate rapidly without sustained practice [20]. In contrast, genuine motor learning reflects durable changes in neural organization and motor control strategies that persist over time and generalize to novel contexts [21]. Evidence from perturbation-based training studies indicates substantial inter-individual variability in older adults’ capacity for motor adaptation, with successful adapters demonstrating superior physical capacity and more effective feedforward adjustments [7]. This heterogeneity suggests that while targeted visuomotor training may drive meaningful adaptation in responsive individuals [13], the extent to which complex tasks like juggling can overcome deeply ingrained “stiffness”—and the time course required for such reorganization—remains an open empirical question.

The theoretical framework articulated by Nikolai Bernstein provides a powerful lens for understanding motor learning progression and its application to postural control in aging populations [22]. Bernstein’s seminal theory posits that motor skill acquisition unfolds through distinct stages: an initial phase characterized by “freezing” of degrees of freedom—wherein learners rigidly constrain joint movements to simplify the control problem—followed by progressive “freeing” of degrees of freedom as expertise develops, permitting flexible, context-appropriate movement solutions [22–24]. This framework maps directly onto the “stiffness” strategies observed in older adults: increased co-contraction effectively “freezes” degrees of freedom at multiple joints [6, 25], reducing the dimensionality of the postural control problem while simultaneously sacrificing adaptability [8, 9]. Conversely, successful aging and effective balance interventions might be characterized by a capacity to “free” degrees of freedom [26, 27], enabling controlled variability that enhances responsiveness to perturbations [28].

Crucially, Bernstein’s theory necessitates a more nuanced interpretation of postural sway metrics than conventional clinical approaches afford [29]. Traditional clinical wisdom equates increased sway path length with instability and elevated fall risk [30]; however, when increased sway path area co-occurs with high precision (minimal radial error from a target position), this pattern may instead reflect an “active monitoring” strategy—deliberate exploratory movements that probe stability boundaries and facilitate rapid corrective responses [31, 32]. Such “active monitoring” stands in stark contrast to rigid, minimal-sway postures that, while superficially stable, may signal “frozen” degrees of freedom and compromised capacity for dynamic adaptation [28]. Empirical evidence demonstrates that adjustments in stiffness, co-contraction, sensorimotor delay, and feedback gains collectively shape postural responses with aging [8, 10], revealing a complex trade-off between immediate stability and the flexibility inherent in “freeing” movement degrees of freedom [7, 9].

An additional dimension of this reorganization concerns the cognitive cost of postural control [4]. Motor learning theory, particularly the three-stage model proposed by Fitts and Posner, predicts that newly acquired motor skills initially demand substantial attentional resources during the cognitive and associative phases, with automatization emerging only after extensive practice in the autonomous phase [33]. In older adults, the cognitive demands of postural control are often assessed via dual-task paradigms [34], wherein a concurrent cognitive task (e.g., serial subtraction) reveals the attentional resources allocated to balance maintenance [4]. The dual-task cost (DTC)—the performance decrement under dual-task conditions relative to single-task baseline—serves as an index of control automaticity [35]. Paradoxically, older adults sometimes exhibit negative DTC values (improved postural stability under cognitive load), a phenomenon attributed to the “supra-postural” effect wherein cognitive tasks reduce maladaptive conscious interference with automatic postural processes [36, 37]. However, when “stiffness” strategies dominate, cognitive loading may exacerbate co-contraction [11], further reducing adaptability [7].

Thus, interventions promoting neuroplasticity and motor relearning face a dual challenge: they must not only foster a transition from ”frozen,” stiffness-dominated control toward freed, actively monitored postural strategies [24, 38, 39], but also facilitate the automatization of these new strategies to minimize cognitive cost [33]. Whether a 4-week visuomotor training intervention is sufficient to achieve both biomechanical reorganization and cognitive efficiency—or whether these processes dissociate temporally—remains unclear.

From a geroscience perspective, the age-related shift toward rigid postural control is often viewed as a consequence of reduced physiological complexity and diminished adaptive capacity (homeostenosis) [40]. However, a critical question remains: does this “stiffness” strategy represent a permanent loss of motor function (structural deficit) or a reversible compensatory mechanism adopted to minimize neural processing costs? If the latter is true, then the aging motor system may retain a latent “motor reserve”—a reservoir of potential plasticity that can be re-engaged by sufficiently challenging stimulation [15, 41]. Differentiating between inevitable decline and modifiable functional adaptations is crucial for defining the limits of restorative rehabilitation in older adults.

Drawing on Bernstein’s theory of “freezing” and “freeing” degrees of freedom, we hypothesized that juggling training would induce a qualitative shift in postural control strategy among older adults: from a static “stiffness” strategy to a dynamic “active monitoring” strategy. Specifically, we predicted that the intervention would lead to a functional reorganization of sway dynamics, characterized by increased exploratory corrective movements (reflected in elevated sway path metrics) without a concurrent increase in postural instability, whereas the passive control condition would reflect continued reliance on rigid postural constraints. Furthermore, we hypothesized that this biomechanical reorganization would be accompanied by a reduction in DTC, indicating progression toward automatization of the newly acquired control strategy.

## Methods

### Study design

This study is a secondary analysis of a randomized crossover design (AB/BA sequence) described in previous work regarding the clinical efficacy of juggling training [42]. The intervention protocol consisted of a 4-week visuomotor training program (cascade juggling with three balls) performed 3 days a week for 45 min. In the crossover design, participants underwent both the training period and a control (passive) period separated by a 4-week washout. Full details of the instructional methodology and compliance monitoring are reported elsewhere [42, 43]. The 4-week intervention duration was selected to target early-stage neural adaptations and functional strategy reorganization, which typically precede significant morphological changes or full motor automatization [44]. This timeframe is sufficient to induce measurable neuroplasticity in older adults, as evidenced by previous studies on visuomotor training [15], allowing us to capture the transition from rigid stiffness to adaptive motor control.

The study was conducted in accordance with the Declaration of Helsinki 2013 and approved by the Ethics Committee of Poznan University of Medical Sciences (No. 106/21, date: 04.02.2021) and was registered retrospectively at ClinicalTrials.gov (NCT06108713).

### Participants

Twenty-six community-dwelling older adults were recruited. For the purpose of this secondary mechanistic analysis, data from one participant were excluded due to incomplete posturographic recordings (final *n* = 25; mean age: 70 ± 4).

The detailed inclusion/exclusion criteria and the randomization procedure are available in the primary publication [42]. In short, participants were healthy, functionally independent seniors with no history of neurological or orthopedic disorders affecting balance. All participants signed a written informed consent form and were informed that they could withdraw from participation at any time.

The sample size was determined by the primary clinical study [42] and was not specifically calculated for the mechanistic variables used in this secondary analysis. However, the use of a crossover design significantly increases statistical power by allowing within-subject comparisons, mitigating the limitations of the relatively small sample size.

### Data processing

Postural sway data were acquired using an AMTI AccuGait™ force platform (AMTI PJB-101, Watertown, MA) utilizing Balance Trainer software with a sampling frequency of 100 Hz. Consistent with standard posturographic recommendations and the methodology detailed in our primary clinical report [42] and standard posturographic recommendations, raw CoP signals were low-pass filtered using a procedure with a cut-off frequency of 10 Hz to eliminate high-frequency noise [45]. Regarding the experimental conditions, participants originally performed balance trials under both single-task (quiet standing) and dual-task (counting numbers during standing) protocols. Each protocol required standing freely with eyes open for 60 s.

The analysis was conducted in two complementary stages. First, to assess the fundamental reorganization of the postural control strategy (Bernstein’s hypothesis), outcome measures were calculated as composite scores averaged across task conditions (single and dual). This pooling approach was chosen to capture the robust, dominant motor pattern acquired through training, independent of momentary task demands. Second, to explicitly quantify the attentional resources required to maintain this strategy, we calculated the DTC based on the separated single and dual-task trials. This distinction allows for a simultaneous evaluation of motor strategy qualitative changes (Stage 1) and their cognitive efficiency (Stage 2).

To test the hypothesis of a shift from “stiffness” to “active monitoring,” we operationalized Bernstein’s concepts using a multidimensional set of CoP parameters. Standard geometric measures, including the 95% confidence ellipse area (Area95) and directional variability (medio-lateral, Xsd; anterior-posterior, Ysd), were computed to quantify spatial confinement. Dynamic metrics included mean sway velocity (Vavg), radial standard deviation (RDLsd) as a proxy for central precision, and path density (PD)—calculated as the ratio of path length to sway area (*cm*^*−1*^). In this framework, “stiffness” (“freezing”) is operationally defined as low spatial variability coupled with low velocity, whereas “unfreezing” (active monitoring) is defined as a concurrent increase in exploratory behavior (higher PD and Vavg) maintained within a stable or reduced spatial zone (stable.lower RDLsd and Area95).

### Statistical analysis

All statistical computations were performed within the R computing environment (Version 4.5.2, R Foundation for Statistical Computing, Vienna, Austria). Descriptive statistics were calculated as estimated marginal means (EMM) with 95% confidence intervals (95% CI).

To account for the repeated measures design and individual variability in baseline postural strategies, Linear mixed-effects models (LMM) were employed using the lme4 package [46]. Unlike traditional repeated-measures ANOVA used in the primary study [42], LMMs offer superior robustness in handling missing data points and modeling subject-specific intercepts. Significance of fixed effects was evaluated using type III Wald *F*-test with Satterthwaite’s method for degrees of freedom approximation.

The modeling strategy followed a two-step procedure to ensure the validity of the crossover design. First, a preliminary full-interaction model was constructed, including the three-way interaction *Time* × *Condition* × *Group* to explicitly test for potential carry-over effects (i.e., whether the intervention effect depended on the sequence order). Since no statistically significant carry-over effects were detected (*p* > 0.05), the three-way interaction term was removed to maximize statistical power.

The final model was specified with *Condition* (*C*: juggling vs. control), *Time* (*T*: pre vs. post), and their interaction (*C* × *T*) as fixed effects. Additionally, the factor *Group* (G: Sequence order: AB vs. BA) was retained as a main effect to statistically control for baseline differences observed between the sequences. The participant ID was included as a random intercept to control for between-subject variance (1|ID).

The primary outcome of interest was the two-way interaction effect (*C* × *T*), which indicated whether the magnitude of change over time differed between the active training and passive control periods. Following the omnibus test, post hoc pairwise comparisons were conducted using the emmeans package [47] to evaluate simple main effects (e.g., pre vs. post changes within each condition).

Model assumptions were verified using the DHARMa package [48] for residual diagnostics with visual inspection of residual plots, supported by Levene’s test, indicating no deviations from homoscedasticity or significant outliers. No significant violations were detected that would require data transformation. The threshold for statistical significance was set at alpha = 0.05.

Given the exploratory nature of the mechanistic analysis and the interdependence of posturographic parameters derived from the same CoP trajectory, no correction for multiple testing across dependent variables was applied to avoid inflating Type II error rates; however, post hoc pairwise comparisons within each model were adjusted using Holm’s method.

## Results

The LMM demonstrated satisfactory fit to the data, with conditional *R*^2^ (*R*^2^_C_) values ranging from 0.55 (PD) to 0.76 (Area95), confirming the importance of including subject-specific random intercepts to account for high inter-individual variability in postural control.

**Fig. 1:**
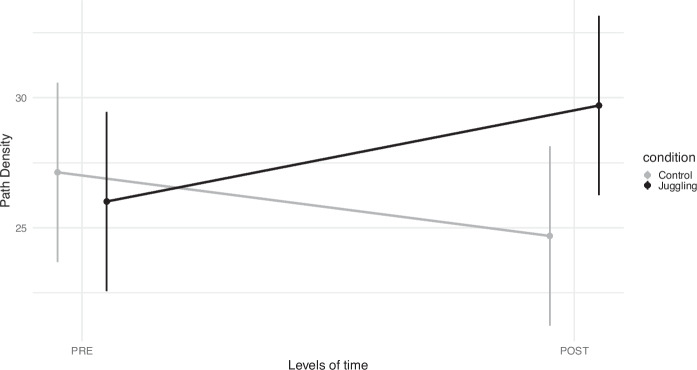
*Time* × *Condition* interaction for path density. The divergent trajectories highlight a fundamental shift in postural strategy. The significant increase in the Juggling condition reflects re-engaged exploratory behavior (restored motor flexibility), whereas the progressive decline in the Control condition indicates an increased reliance on rigid postural constraints (“stiffness”/“freezing”)

The LMM revealed a significant *Time* × *Condition* interaction for PD (*p* = 0.014, *η*_p_^2^ = 0.08). Specifically, in the juggling condition, PD increased from 26.0 to 29.7 cm^−1^ (*p* = 0.035), whereas in the control condition, a reduction from 27.1 to 24.7 was observed (*p* = 0.159). For details, see Fig. 1.

**Fig. 2:**
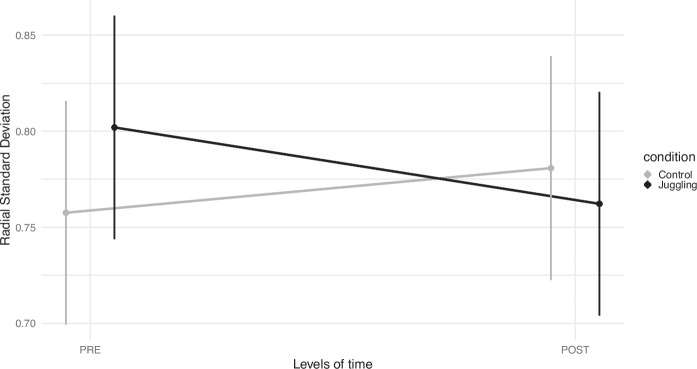
*Time* × *Condition* interaction for radial standard deviation. A reduction in RDLsd indicates improved central precision (stability). The juggling condition demonstrated enhanced precision despite increased movement activity (see Fig. 1), confirming the efficiency of the “active monitoring” strategy. In contrast, the control condition showed a slight deterioration in precision, consistent with a maladaptive “stiffness” response

Similarly, a significant interaction was found for RDLsd (*p* = 0.040, *η*_p_^2^ = 0.06). Visuomotor training improved central precision, reducing the RDLsd from 0.80 to 0.76 cm, contrasting with the slight deterioration observed in the control condition (increase from 0.76 to 0.78 cm). While post hoc pairwise comparisons for RDLsd did not reach the threshold for significance (juggling, *p* = 0.066; control, *p* = 0.278), the significant interaction confirms that the trajectory of change differed significantly between the two conditions (see Fig. 2). However, this finding should be interpreted with caution. Given the exploratory nature of this secondary analysis and the testing of multiple CoP-derived metrics without across-model adjustment for family-wise error, this result warrants replication in larger, prospectively powered cohorts.

In terms of directional stability, no significant interaction effects were observed for the Xsd or Ysd planes (*p* > 0.05). However, a significant main effect of *Group* was found for Ysd (*p* < 0.001, *η*_p_^2^ = 0.41), indicating that participants in the BA sequence were characterized by significantly better baseline stability compared to the AB sequence. This main effect of *Group* was also observed for PD (*p* = 0.043, *η*_p_^2^ = 0.17) and Area95 (*p* = 0.008, *η*_p_^2^ = 0.27), with consistently better results for the BA sequence. No other statistically significant effects were observed. Detailed results of the LMM analysis are presented in Table 1.

**Table 1:** Summary of linear mixed model analysis for posturographic variables across *Condition*, *Time*, and *Group* sequence

Variable	Condition	Time	EMM	95% CI	SE	*F*_(df)_	*p*-value	Model fit
Xsd [*cm*]	Control	Pre	0.93	[0.85, 1.00]	0.04	***C*:** 0.06_(1,72)_ ***T:*** 0.61_(1,72)_ ***C*** **× **T**:** 2.43_(1,72)_ ***G: ***3.51_(1,23)_	***C*:** 0.799 ***T:*** 0.438 ***C*** **× **T**:**0.124 ***G: ***0.074	***R***^**2**^_**M**_**:** 0.102 ***R***^**2**^_**C**_**:** 0.666
		Post	0.87	[0.79, 0.95]				
	Juggling	Pre	0.90	[0.81, 0.98]				
		Post	0.92	[0.83, 1.00]				
Ysd [*cm*]	Control	Pre	1.08	[1.01, 1.15]	0.04	***C*:** 0.02_(1,72)_ ***T*:** 0.65_(1,72)_ ***C* × **T**:** 2.96_(1,72)_ ***G: ***16.11_(1,23)_	***C*:** 0.889 ***T*:** 0.422 ***C* × **T**:** 0.090 ***G*: < **0.001*	***R***^**2**^_**M**_**:** 0.308 ***R***^**2**^_**C**_**:** 0.683
		Post	1.02	[0.95, 1.09]				
	Juggling	Pre	1.03	[0.96, 1.11]				
		Post	1.06	[0.98, 1.13]				
PD [*cm*^*−1*^]	Control	Pre	26.0	[22.6, 29.5]	1.72	***C*:** 2.57_(1,72)_ ***T*:** 0.26_(1,72)_ ***C* × *T*:** 6.38_(1,72)_ ***G: ***4.55_(1,23)_	***C*:** 0.113 ***T*:** 0.609 ***C* × **T**: **0.014* ***G*:** 0.043*	***R***^**2**^_**M**_**:** 0.138 ***R***^**2**^_**C**_**:** 0.547
		Post	29.7	[26.3, 33.1]				
	Juggling	Pre	27.1	[23.7, 30.6]				
		Post	24.7	[21.2, 28.1]				
RDLsd [*cm*]	Control	Pre	0.80	[0.74, 0.86]	0.03	***C*:** 0.74_(1,72)_ ***T*:** 0.30_(1,72)_ ***C* × **T**:** 4.39_(1,72)_ ***G: ***4.55_(1,23)_	***C*:** 0.393 ***T*:** 0.584 ***C* × **T**:** 0.040* ***G: ***0.215	***R***^**2**^_**M**_**:** 0.063 ***R***^**2**^_**C**_**:** 0.726
		Post	0.76	[0.70, 0.82]				
	Juggling	Pre	0.76	[0.70, 0.82]				
		Post	0.78	[0.72, 0.84]				
Area95 [*cm*^*2*^]	Control	Pre	43.9	[37.5, 50.3]	3.17	***C*:** 0.01_(1,72)_ ***T:*** **1**.79_(1.72)_ ***C* × **T**:** 2.00_(1,72)_ ***G: ***8.52_(1,23)_	***C*:** 0.903 ***T*:** 0.185 ***C* × **T**:** 0.162 ***G*:** 0.008*	***R***^**2**^_**M**_**:** 0.217 ***R***^**2**^_**C**_**:** 0.759
		Post	39.2	[32.8, 45.7]				
	Juggling	Pre	41.7	[35.3, 48.1]				
		Post	41.8	[35.4, 48.3]				
Vavg [*cm/s*]	Control	Pre	2.48	[2.35, 2.61]	0.07	***C*:** 2.48_(1,72)_ ***T*:** 0.01_(1,72)_ **C ×** ***T:*** 0.49_(1,72)_ ***G: ***3.08_(1,23)_	***C*:** 0.120 ***T*:** 0.912 ***C* × **T**:** 0.486 ***G: ***0.093	***R***^**2**^_**M**_**:** 0.093 ***R***^**2**^_**C**_**:** 0.682
		Post	2.46	[2.33, 2.59]				
	Juggling	Pre	2.51	[2.38, 2.65]				
		Post	2.55	[2.41, 2.68]				

* statistical significance, EMM estimated marginal means, *95% CI* 95% confidence interval, *SE* standard error, *F*_(df)_ *F*-test value with degrees of freedom; model fit assessed via by Nakagawa’s *R*^2^ (*R*^2^_M_, marginal *R*-squared; *R*^2^_C_, conditional *R*-squared); *Xsd/Ysd* standard deviation of center of pressure in medio-lateral/anterior–posterior direction, *PD* ratio of path length to area (path density), *RDLsd* radial standard deviation, *Area95* 95% confidence ellipse area, *Vavg* mean sway velocity, *C* main effect *Condition*, *T* main effect *Time*, *C* × *T* interaction effect of *Time* and *Condition*, *G* main effect *Group* (sequence)

Analysis of the DTC for Vavg did not reveal a statistically significant *Time* × *Condition* interaction (*p* = 0.080). In terms of EMM, the control condition showed a decrease from −0.8% to −4.7%. In contrast, the juggling condition recorded mean values moving from −0.6% to + 0.5%. Detailed results are presented in Table 2.

**Table 2:** Summary of linear mixed model analysis for DTC variable across *Condition*, *Time*, and *Group* sequence

Variable	Condition	Time	EMM	95% CI	SE	*F*_(df)_	*p*-value	Model fit
DTC [%]	Control	Pre	− 0.81	[− 3.86, 2.24]	1.53	***C*:** 3.66_(1,72)_ ***T*: 1**.01_(1,72)_ ***C* × *T*:** 3.15_(1,72)_ ***G***: 0.26_(1,23)_	***C*:** 0.06 ***T*:** 0.319 ***C* × *T*:** 0.080 ***G***: 0.612	***R***^**2**^_**M**_**:** 0.066 ***R***^**2**^_**C**_**:** 0.217
		Post	− 4.65	[− 7.70, − 1.60]				
	Juggling	Pre	− 0.62	[− 3.67, 2.43]				
		Post	0.45	[− 2.60, 3.49]				

*EMM* estimated marginal means, *95% CI* 95% confidence interval, *SE* standard error, *F*_(df)_ *F*-test value with degrees of freedom, *DTC* dual-task cost, *C* main effect of *Condition*, *T* main effect of *Time*, *C* × *T* interaction effect of *Time* and *Condition*, *G* main effect *Group* (sequence)

## Discussion

The primary aim of this study was to determine whether complex visuomotor training (juggling) induces a qualitative reorganization of postural control strategies in older adults and, critically, whether such reorganization translates into reduced cognitive cost—a hallmark of motor skill automatization [20, 33]. Our results provide insight into motor adaptation in aging, suggesting a dissociation between biomechanical reorganization and cognitive efficiency. Consistent with our operational definition, the intervention successfully shifted participants from a maladaptive “stiffness” strategy to a functional “active monitoring” strategy: this was evidenced quantitatively by significantly increased PD coupled with improved central precision (reduced RDLsd). However, contrary to our expectations, this physical reorganization was not accompanied by a statistically significant reduction in DTC (*p* = *0.08*), pointing to a complex, temporally extended relationship between the emergence of new motor patterns and their automatization [21]. This dissociation carries important theoretical and practical implications for understanding motor learning in aging populations [19] and for designing effective balance rehabilitation programs [14].

A central and novel finding of this work is the divergent trajectory of kinematic variables in the intervention versus control conditions, a pattern that aligns closely with Bernstein’s theory of “freezing” and “freeing” degrees of freedom [22, 24]. Participants in the control period exhibited a progressive reduction in PD over the 4-week interval. We interpret this reduction as a defensive strategy aimed at minimizing postural disturbances through increased co-contraction and joint stiffening [6, 7]. This “freezing” response is consistent with the well-documented tendency of older adults to prioritize immediate stability over adaptability [7, 11], particularly in the absence of targeted intervention [13]. In stark contrast, juggling training reversed this trajectory, producing a significant increase in PD. Importantly, this elevation in movement activity did not signify instability; rather, it was paired with a significant reduction in RDLsd, indicating that participants were executing more frequent corrective movements within a tightly constrained central zone.

**Fig. 3:**
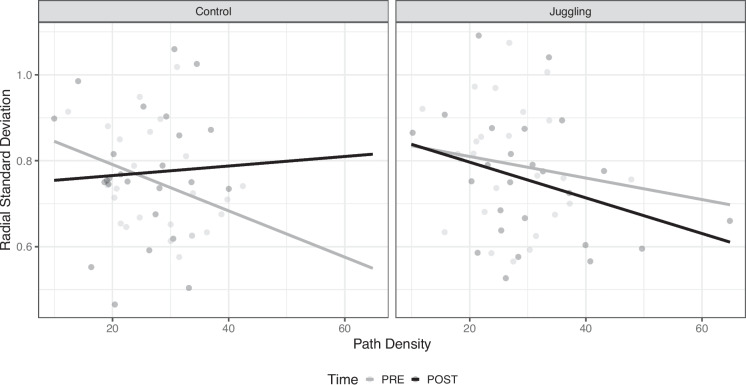
The kinematic signature of the “active monitoring” strategy. The regression lines illustrate the functional relationship between sway activity and stability. In the juggling condition (right panel), the negative slope at post-test confirms that increased exploratory behavior (higher path density) is effectively utilized to constrain spatial instability (lower radial standard deviation)—fulfilling the definition of “unfreezing.” In contrast, the control condition (left panel) at post-test exhibits a flattening of this relationship, indicating that sway activity is reduced (stiffness) and no longer translates into enhanced central precision

Functionally, this kinematic signature (see Fig. 3)—increased exploratory behavior (higher PD) coupled with enhanced precision (lower RDLsd)—fulfills our operational definition of “unfreezing” [32]. From a control perspective, this suggests the central nervous system actively deploys high-frequency, small-amplitude corrections rather than relying on passive mechanical stiffness to maintain equilibrium [12]. From a Bernsteinian perspective, this represents a “freeing” of degrees of freedom [22, 29]: participants gained the capacity to modulate joint movements fluidly and context-sensitively, probing stability boundaries and making rapid adjustments as needed [28]. Such a strategy is functionally superior to rigid stiffness, as it preserves the adaptability required to respond effectively to unexpected perturbations [7]—a critical capability for fall prevention in real-world environments [5]. The fact that this reorganization occurred within a relatively brief 4-week training period underscores the retained neuroplasticity of the aging motor system [15, 19] and the potency of complex visuomotor tasks as catalysts for motor adaptations [16]. However, it remains to be determined whether this reorganization reflects durable motor learning or a transient performance enhancement, as the present study did not include a retention test.

Despite the clear biomechanical evidence for strategy reorganization, the cognitive cost analysis revealed an intriguing dissociation. In the control condition, the imposition of a concurrent cognitive task (dual-task condition—counting numbers) exacerbated the stiffness response, driving DTC into negative values—a pattern consistent with the “supra-postural” effect [36, 37], wherein cognitive loading paradoxically reduces maladaptive conscious interference but simultaneously amplifies co-contraction in individuals relying on “stiffness” strategies [11]. Following juggling training, we observed a trend toward normalization of DTC (a shift toward positive values, indicating reduced reliance on stiffness under cognitive load), yet this change failed to reach statistical significance.

**Fig. 4:**
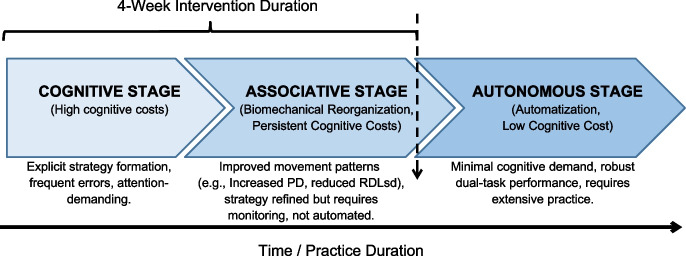
Conceptual representation of motor learning stages based on the Fitts and Posner model, illustrating that the 4-week intervention period (bracket) was sufficient for biomechanical reorganization (associative stage) but insufficient for achieving full automatization (autonomous stage). This framework explains the observed dissociation between motor and cognitive outcomes: while participants successfully acquired the “active monitoring” strategy (improved stability), the execution of this new strategy still requires significant attentional resources (high cognitive cost), preventing a reduction in DTC

This finding—successful motor reorganization without concomitant automatization—can be interpreted through the lens of Fitts and Posner’s three-stage model of motor learning [33]. According to this framework, skill acquisition progresses from a cognitive phase (characterized by explicit, attention-demanding execution and frequent errors) through an associative phase (wherein movement patterns become more consistent and refined, but still require attentional monitoring) to an autonomous phase (marked by automaticity, minimal cognitive demand, and robust performance under dual-task conditions) [33, 49]. Our results suggest that 4 weeks of juggling training was sufficient to advance participants through the cognitive phase—evidenced by their successful acquisition of a new postural control strategy—and into the associative phase, wherein the strategy is structurally correct and biomechanically effective but not yet fully automated (Fig. 4) [13]. The persistent cognitive cost reflects the continued engagement of attentional resources for online monitoring and refinement of the newly acquired “active monitoring” strategy.

This interpretation is consistent with the broader motor learning literature, which indicates that automatization—the hallmark of the autonomous phase—typically requires extensive practice over prolonged periods, often months rather than weeks [20, 21]. In younger adults, the transition from associative to autonomous control is facilitated by repeated exposure to varied task conditions, which promotes the consolidation of motor memories and the development of robust, generalized motor programs [21]. In older adults, this process may be further protracted due to age-related declines in neural plasticity [19], reduced processing speed [50], and diminished capacity for motor memory consolidation [51]. Thus, while our 4-week intervention successfully induced a qualitative shift in postural control strategy, achieving full automatization—and the associated liberation of attentional resources—likely requires a longer training duration or more intensive practice regimen (dose response effect) [14].

However, beyond training dosage, we must also consider the role of task specificity. While juggling places high demands on visuomotor processing, the transfer of these gains to the specific cognitive dual-task used in assessment (number count) may be limited compared to the trained task itself [52]. Regarding functional translation, we hypothesize that the observed “unfreezing” represents a necessary biomechanical prerequisite for stability, but its real-world benefit—specifically regarding fall risk reduction—may only fully emerge once the strategy becomes automatized and less attentionally demanding. To rigorously test this temporal dissociation model, future research should employ longitudinal designs of extended duration (> 8 weeks) and incorporate neurophysiological measures (e.g., fNIRS or EEG) to explicitly track the time-course of neural efficiency alongside kinematic reorganization.

The dissociation observed in this study—successful motor reorganization without significant cognitive cost reduction—carries important theoretical implications for understanding motor learning in aging populations [19]. First, it challenges the implicit assumption, prevalent in much of the balance training literature, that improvements in postural control strategy necessarily translate into immediate reductions in cognitive demand [4]. Our findings suggest that these processes can dissociate temporally, with biomechanical changes preceding cognitive efficiency gains. This temporal dissociation aligns with hierarchical models of motor control [22, 53], which posit that motor skill acquisition involves distinct levels of neural organization: a lower level concerned with the kinematic and kinetic parameters of movement execution, and a higher level responsible for strategic planning, attentional allocation, and cognitive-motor integration [53]. Visuomotor training may initially target the lower level, reorganizing the motor synergies and control policies governing postural sway [28], while higher-level automatization—reflected in reduced cognitive cost—emerges only after these new patterns have been extensively practiced and consolidated [21, 33].

Furthermore, these findings support the application of the concept of “motor reserve” [41, 54]—the ability of older adults to rapidly “unfreeze” their posture suggests that age-related stiffness is not an irreversible decline, but a modifiable state. The intervention likely re-engaged latent neural plasticity, suggesting that the aging motor system retains the capacity for reorganization even if full automatization requires more time [15, 19].

Second, our results highlight the importance of distinguishing between motor competence (the ability to execute a skill effectively) and motor automaticity (the ability to execute a skill with minimal cognitive demand) [20]. In clinical and rehabilitation settings, assessments of balance often focus exclusively on kinematic or kinetic outcomes (e.g., sway magnitude and CoP velocity) without evaluating cognitive cost [30]. However, our findings suggest that such assessments may provide an incomplete picture of functional improvement. An older adult who has acquired a biomechanically superior postural strategy but has not yet automatized it remains at risk for falls in cognitively demanding real-world situations (e.g., walking while conversing and navigating crowded environments), where attentional resources are divided among multiple tasks [34, 35]. Thus, comprehensive evaluation of balance training outcomes should incorporate dual-task paradigms to assess not only what participants *can* do but also the cognitive cost of doing it [4].

## Clinical and practical implications

From a clinical perspective, our findings underscore the necessity of extended intervention durations in gerontological rehabilitation programs aimed at improving postural control [14]. While a 4-week juggling training program successfully reorganized participants’ postural control strategies at the biomechanical level—shifting them from maladaptive “stiffness” to functional “active monitoring”—this duration was insufficient to achieve full automatization, as evidenced by the persistent (albeit reduced) cognitive cost. To translate improvements in movement quality into lasting savings of attentional resources, balance training interventions for older adults may need to extend over periods of 2 to 3 months or longer [20], with progressive increases in task complexity and variability to promote consolidation and generalization [21].

Moreover, our results suggest that individualized intervention design—tailored to participants’ baseline stability and cognitive-motor profiles—may enhance training efficacy [13]. Participants with poorer baseline stability and higher initial cognitive costs may benefit most from intensive visuomotor training, as they have greater potential for improvement and are less constrained by ceiling effects [55, 56]. Conversely, individuals with already-high baseline performance may require alternative or supplementary interventions (e.g., perturbation-based training and dual-task practice) to achieve further gains [7].

## Limitations

Several limitations of this study warrant consideration. First, the relatively small sample size (*n* = 25) was determined by the primary clinical trial and was not prospectively powered for the mechanistic secondary endpoints analyzed here. Consequently, the statistical power to detect subtle changes in cognitive-motor interactions (DTC) may have been limited. Although the reduction in DTC did not reach the threshold for statistical significance, this result points to a potential Type II error; therefore, these findings should be interpreted as exploratory and hypothesis-generating, providing effect size estimates for future, fully powered longitudinal studies. Second, although we employed a crossover design, we observed a significant sequence effect in baseline stability variables. While our statistical models controlled for this factor, residual confounding cannot be entirely ruled out. Third, our assessment was limited to laboratory-based postural sway metrics and a single dual-task paradigm (serial counting). It is possible that this specific cognitive task lacked sufficient complexity to impose a high attentional load on this relatively healthy cohort, potentially leading to ceiling effects that masked improvements in automatization. Furthermore, while the juggling protocol involved progressive motor challenges, the absence of varied dual-task training conditions (e.g., combining motor practice with concurrent cognitive demands) may have limited the generalization of motor gains to the specific cognitive dual-task used in our assessment. Incorporating additional cognitive tasks and real-world balance challenges [5] would provide a more comprehensive evaluation of functional improvement and generalization. Fourth, we did not assess neural correlates of motor learning (e.g., via neuroimaging or electrophysiology), which would offer direct insight into the brain mechanisms underlying the observed behavioral changes [15, 19]. Finally, the absence of a retention test precludes conclusions about the long-term durability of training effects; future work should include follow-up assessments at intervals of several months to evaluate the persistence of both kinematic and cognitive adaptations [21].

## Conclusion

In summary, our results suggest that four weeks of complex visuomotor training (juggling) may facilitate reorganization of the postural control system at the executive level, inducing a transition from a maladaptive “stiffness” strategy to a functionally superior “active monitoring” strategy characterized by increased PD and improved central precision. This finding supports the application of Bernstein's theory of “freezing” and “freeing” degrees of freedom and highlights the retained neuroplasticity of the aging motor system. However, the absence of a statistically significant reduction in DTC indicates that this biomechanical reorganization is not synonymous with immediate automatization. Rather, participants appear to have progressed into the associative phase of motor learning, wherein the new control strategy is structurally sound but still requires attentional resources for online monitoring and refinement. This dissociation between motor pattern change and cognitive efficiency has important implications for both theory and practice: it suggests that biomechanical and cognitive adaptations may follow distinct time courses, and it highlights the need for extended intervention durations—likely 2 to 3 months or longer—to achieve full automatization and lasting reductions in cognitive cost. Future research should explore the neural mechanisms underlying this dissociation, evaluate longer training protocols, and assess the generalization of training effects to real-world functional tasks and fall risk reduction.

## Data Availability

The data supporting the findings of this study are available upon reasonable request. Request to access the datasets will be reviewed by corresponding author Jakub Malik at Poznan University of Physical Education, in accordance with intellectual property protections, confidentiality agreements, and applicable ethical regulations.
